# Lymphocyte phosphatase-associated phosphoprotein proteoforms analyzed using monoclonal antibodies

**DOI:** 10.1038/cti.2015.22

**Published:** 2015-10-09

**Authors:** Alexander Filatov, Natalia Kruglova, Tatiana Meshkova, Dmitriy Mazurov

**Affiliations:** 1 Laboratory of Immunochemistry, Institute of Immunology, Moscow, Russia; 2 Faculty of Biology, Lomonosov Moscow State University, Moscow, Russia

## Abstract

Phosphatase CD45 regulates the activation of lymphocytes by controlling the level of receptor and signal molecule phosphorylation. However, it remains unknown which molecules mediate the phosphatase activity of CD45. A candidate for such a molecule is a small transmembrane adapter protein called lymphocyte phosphatase-associated phosphoprotein (LPAP). LPAP forms a supramolecular complex that consists of not only CD45 molecule but also CD4 and Lck kinase. The function of LPAP has not been defined clearly. In our study, we determined the pattern of LPAP expression in various cell types and characterized its proteoforms using new monoclonal antibodies generated against the intracellular portion of the protein. We show that LPAP is a pan-lymphocyte marker, and its expression in cells correlates with the expression of CD45. The majority of T, B and NK cells express high levels of LPAP, whereas monocytes, granulocytes, monocyte-derived dendritic cells, platelets and red blood cells are negative for LPAP. Using one- and two-dimensional protein gel electrophoresis, we demonstrate that LPAP has at least four sites of phosphorylation. The resting cells express at least six different LPAP phosphoforms representing mono-, di- and tri-phosphorylated LPAP. T and B cells differ in the distribution of the protein between phosphoforms. The activation of lymphocytes with PMA reduces the diversity of phosphorylated forms. Our experiments on Lck-deficient Jurkat cells show that Lck kinase is not involved in LPAP phosphorylation. Thus, LPAP is a dynamically phosphorylated protein, the function of which can be understood, when all phosphosites and kinases involved in its phosphorylation will be identified.

The human lymphocyte phosphatase-associated phosphoprotein (LPAP) is a type I transmembrane protein with a predicted MW of 19 kDa.^1^ The murine homolog of human LPAP is often designated in the literature as a CD45-associated protein (CD45-AP).^2^

LPAP does not belong to any known protein family and has no protein homologs. Although it was described long ago, the function of this protein remains elusive. Multiple observations suggest that LPAP has an important role in the regulation of lymphocyte activation. First, LPAP is tightly associated with the phosphatase CD45, which is a key regulator of T- and B-lymphocyte signaling. Approximately 75% of the total CD45 and LPAP proteins in cells are present in the form of a supramolecular complex.^3^ Second, LPAP is a phosphorylated protein, and the level of its phosphorylation changes upon the activation of lymphocytes. In particular, it has been shown that Ser99 in LPAP undergoes phosphorylation after the antigenic stimulation of lymphocytes.^4^ In contrast to the results obtained *in vitro,* three independent laboratories that generated CD45-AP knockout mice reported no pronounced phenotype in these mice; neither significant defects in the immune system nor other morphological changes have been detected.^5, 6, 7^ Thus, the function of LPAP *in vivo* has not been demonstrated.

The protein structure and topology are not resolved for LPAP and have been described only in theoretical models. LPAP starts with a signal peptide (amino acid 1–20), which is cleaved during protein maturation. The hydrophobic portion of the protein (amino acid 31–53) represents the transmembrane domain. Thus, the extracellular part of LPAP consisting of ~10 amino acids is very short. The intracellular portion of LPAP contains a WW-like domain^8^ and a glutamic and aspartic acid rich domain called the ‘acidic domain' that mediates the interaction of LPAP with Lck kinase.^9^ Data indicate that murine CD45-AP can be expressed in two isoforms.^10^ For both human and murine LPAP, several potential phosphorylated forms of the protein have been reported based on electrophoretic protein mobility shift assays. In particular, the LPAP proteoforms with molecular weights (MWs) of 32 and 29 kDa were detected in the resting human Jurkat T-cell line, whereas the LPAP proteoforms with MWs of 30 and 31 kDa were observed in Jurkat cells activated with phorbol myristate acetate (PMA).^1^ Nevertheless, a detailed analysis of various proteo- and phosphoforms of LPAP has not yet been conducted.

To some extent, the difficulties in defining LPAP structure and function are because of the absence of monoclonal antibodies (mAbs) specific for the different domains of this protein; only polyclonal antibodies against LPAP generated during immunization with specific peptides^1^ or the entire cytoplasmic domain^11^ of LPAP have been reported. Previously, we developed a panel of mAbs raised by the immunization of mice with recombinant LPAP.^12^ The generated mAbs recognize three non-overlapping epitopes in the cytoplasmic domain of LPAP; mAb CL3 binds to the epitope between amino acid 184 and the C terminus, CL4 is specific for the epitope between amino acid 122 and 137, and CL7 recognizes LPAP around Thr113. mAb CL3, CL4 and CL7 were presented on and tested by the Tenth International workshop on Human Leukocyte Differentiation Antigens. In the current study, we fully characterized the anti-LPAP mAbs CL3, CL4 and CL7 by testing them in immunoprecipitation (IP), western immunoblotting and immunochemistry assays. Using immunofluorescent staining and flow cytometry analysis, we re-evaluated the LPAP expression pattern in various cell types. Using new mAbs and two-dimensional (2D) electrophoretic separation of proteins, we show that depending on cell status (activation or resting) and cell type, LPAP can be detected in at least seven proteoforms. Six of these proteoforms were sensitive to the treatment with calf intestinal phosphatase (CIP) and converted to the most alkaline form of LPAP. These data suggest that proteoforms of LPAP detected with mAbs and 2D electrophoresis mostly represent differently phosphorylated forms (phosphoforms) of LPAP.

## RESULTS

### Analysis of LPAP expression in various cell types

Because mAbs CL3, CL4 and CL7 specifically recognize intracellular portions of LPAP, cells were first permeabilized and then stained with mAb. In preliminary experiments, the indicated antibodies demonstrated an identical pattern of reactivity with cells (data not shown). Hence, in all of the experiments, we used mAb CL7 to show the distribution of LPAP expression in human cells. After cells were stained with primary and corresponding secondary antibody, samples were analyzed by flow cytometry, fluorescence microscopy or light microscopy.

**Figure 1:**
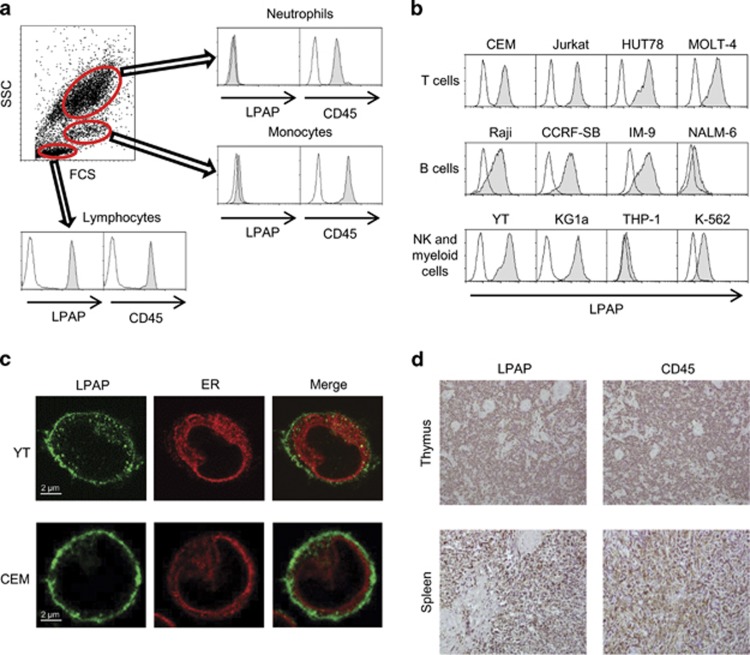
Immunostaining of human primary and transformed cells for LPAP. Flow cytometry analysis of peripheral blood leukocytes (**a**) and haematopoietic cell lines (**b**). Cells were fixed, permeabilized in 0.1% saponin, labeled with an anti-LPAP or an anti-CD45 mAb, followed by incubation with FITC-labeled secondary antibody and analyzed by flow cytometry (shaded plots). Cells stained with isotype-matched irrelevant mouse monoclonal antibody served as a control (open plots). Lymphocytes, monocytes and granulocytes were pre-gated by forward and side scatter. (**c**) Confocal fluorescence images of permeabilized cells stained with anti-LPAP mAb CL7 (green). Endoplasmic reticulum was stained with tetramethylrhodamine-labeled concanavalin A. (**d**) Immunoperoxidase staining of formalin-fixed, paraffin-embedded sections of thymus (upper panel) and spleen (lower panel). Sections were stained with anti-LPAP or anti-CD45 mAb. Original magnification × 150. Representative flow cytometry and microscopy images are shown.

First, we determined the level of LPAP expression in human peripheral blood leukocytes using flow cytometry. As shown in Figure 1a, mAb CL7 was reactive to lymphocytes but not to monocytes and granulocytes. LPAP was also differently expressed in haematopoietic tumor cell lines (Figure 1b). Generally, T-, B- and NK cell lines were positive for LPAP. In contrast to mature cells, early B-cell lines, such as NALM-6, and terminally differentiated myeloma B-cell lines, U266 and RPMI 8226, were negative for LPAP staining. The erythroblastoid (K-562) or acute myeloid (U-937, THP-1 and Mono-Mac-6) cells were also LPAP negative. To determine the intracellular distribution of LPAP, the samples stained with CL7 mAb were examined by confocal microscopy. As shown in Figure 1c, LPAP was predominantly localized in the plasma membrane area but was also found in the cytoplasm or Golgi complex. Generated mAbs were suitable for immunohistochemical staining of LPAP in formalin-fixed tissue sections and exhibited reliable staining patterns in the thymus and spleen and an intensity comparable to the anti-CD45 mAb (Figure 1d). The reactivities of the anti-LPAP mAb with primary haematopoietic cells and tumor cell lines are summarized in Table 1. Thus, using new anti-LPAP mAbs, we determined that the LPAP is expressed in mature lymphocytes but not in cells of the erythroid, myeloid lineage (monocyte and neutrophils), immature or terminally differentiated B cells.

### Expression of LPAP in lymphocytes correlates with the expression of CD45

**Figure 2:**
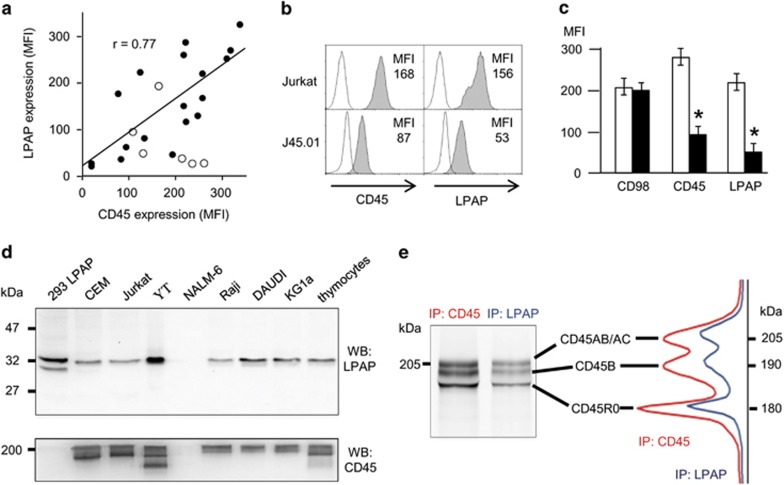
Coexpression of endogenous LPAP and CD45. (**a**) Correlation between the levels of LPAP and CD45 expression in lymphoid cells. The levels of LPAP and CD45 expression in B-, T-, NK cell lines and in myeloid or erythroid cell lines are plotted with filled and open circles, respectively. Pearson's correlation coefficient was calculated only for values depicted with filled circles. (**b**) CD45-deficient Jurkat cell line (J45.01) expressed a reduced level of LPAP. The cell staining including controls was performed as described in Figures 1a and b. (**c**) CD45 knockdown with shRNA reduces the level of LPAP expression. CEM cells were stably transduced with shRNA targeting CD45 (open bars) or phosphoprotein of RSV (control, filled bars), as described in the Methods section. The average values of MFI obtained from three independent experiments with s.d. are shown. *P<0.05 by Student's *t*-test. (**d**) Western blot (WB) analysis of LPAP (upper panel) and CD45 (lower panel) expression in various human cell lines and primary cells. (**e**) HUT78 cells were Cy5 labeled, lysed and analyzed by FIPA as described in the Methods section. CD45 after immunoprecipitation (IP) with CD45 (left lane) or after IP with LPAP (right lane). The densitograms of CD45 bands corresponding to the left lane (red) and right lane (blue) are shown on the right panel. The images (**b**, **d**, and **e**) are representative of three experiments. MFI, mean fluorescence intensity.

Because LPAP is a protein associated with the phosphatase CD45, we compared the pattern of LPAP expression with that obtained for CD45 (Table 1, right column). As shown in Table 1, the levels of expression of these two proteins in different cell types were very similar. To estimate the correlation level between the expression of LPAP and CD45, we plotted the parameters of mean fluorescence intensity obtained for the various cell types stained for LPAP versus mean fluorescence intensity values detected with CD45 staining. As shown in Figure 2a, there was some correlation between the expression of LPAP and CD45 in the same cells (Pearson's correlation coefficient, *r*=0.77). For example, YT and HUT78 T-cell lines expressed the highest level of CD45 and also expressed a high level of LPAP. By contrast, B-cell lines NALM-6, RPMI 8226 and HPB-ALL expressed minimal levels of CD45 and LPAP expression. However, among the cells of the erythroid and myeloid lineages, the correlation between the CD45 and LPAP expression was not obvious. Thus, THP-1, Mono-Mac-6 and K-562 cells expressed high levels of CD45 and low or undetectable levels of LPAP. Interestingly, we did not find any cell type that would express LPAP but would be negative for CD45 expression. Nevertheless, LPAP that was expressed ectopically in HEK293 cells under the constitutive promoter CMV was detected at a high level without the coexpression of CD45 (Figure 2d). These data suggest that the expression of endogenous LPAP is subjected to the regulation that involves CD45 expression in cells.

To evaluate the effect of CD45 expression on the level of LPAP expression in one cell line, we analyzed the expression of these two antigens in Jurkat cells with the normal (wild type, wt) or reduced (J45.01) level of CD45 expression. As shown in Figure 2b, J45.01 cells expressed a twofold lower level of CD45 and threefold lower level of LPAP than that obtained for wt Jurkat cells. In addition, we reduced the level of CD45 expression in Jurkat T cells using short hairpin RNA interference. To achieve this reduction, Jurkat cells were stably transduced with pGIPD lentiviral construct expressing short hairpin RNA, which targeted the CD45 sequence (see details in Methods section). Then, resistant cells were selected in the presence of puromycin. The generated Jurkat cell line with sh-CD45 was stained for CD98 (control), CD45 and LPAP. As demonstrated in Figure 2c, the Jurkat-sh-CD45 cell subline had twofold and more than threefold reduced levels of CD45 and LPAP expression, respectively, compared with the expression levels of these molecules in wt Jurkat cells. Thus, the additional set of experiments on Jurkat cells confirmed the reciprocal pattern of expression detected for CD45 and LPAP proteins.

CD45 phosphatase can be expressed in at least five different isoforms.^13^ Therefore, it was interesting to determine whether the coexpression of LPAP depends on the expression of particular isoforms of CD45. To detect various isoforms of CD45 protein, cell lysates were resolved by 5% SDS–PAGE, blotted and stained with an anti-CD45 mAb (Figure 2d, bottom panel). In parallel, the same lysates were run on 12% SDS–PAGE, blotted and stained for LPAP (Figure 2d, upper panel). As shown in Figure 2d (bottom panel), the high MW isoforms of CD45 were predominantly expressed on B- (Raji and Daudi) and myeloid (KG1a) cell lines, whereas T and NK lymphocytes usually carried the low MW isoforms of CD45. However, no correlation was found between LPAP expression and the expression of CD45 isoforms.

Then, we hypothesized that LPAP can preferentially associate with one or several isoforms of CD45. To test this hypothesis, HUT78 T cells expressing four isoforms of CD45 were Cy5 labeled and lysed in the buffer containing mild detergent Brij97. This detergent preserves the protein complex of CD45 with LPAP. The proteins in lysate were precipitated with either an anti-CD45 mAb or with an anti-LPAP mAb. The samples from two precipitations were resolved on a low percentage SDS–PAGE and visualized by in-gel fluorescence scanning. As shown in Figure 2e, the number and the proportion of CD45 isoforms detected after IP with the anti-CD45 mAb (left lane) and after co-IP with anti-LPAP mAb were identical. These data suggest that LPAP associates with various isoforms of CD45 with the same affinity.

In summary, the expression of endogenous LPAP in lymphocytes is often dependent on the overall level of CD45 expression. However, neither the level of expression nor the association of LPAP with CD45 is determined by the isoforms of CD45.

### LPAP proteoforms detected by one-dimensional electrophoresis

**Figure 3:**
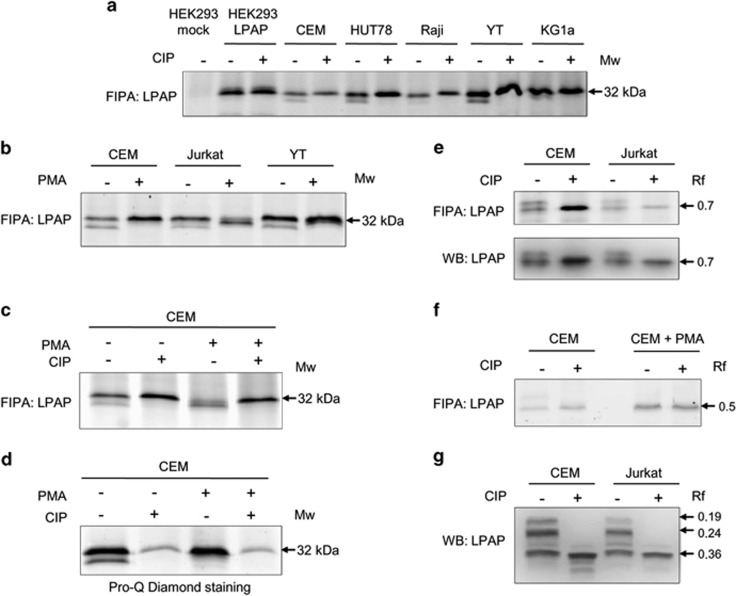
LPAP proteoforms detected by 1D-PAGE. Cells were labeled with fluorescent dye Cy5 and solubilized in lysis buffer. LPAP was immunoprecipitated with CL7 mAb and then treated or not treated with CIP. Samples were run on conventional 18% SDS–PAGE (**a**–**c**) or 10% Phos-tag SDS–PAGE (**e**–**g**). Gels were analyzed with fluorescent gel scanner (FIPA) or by western blotting (WB). (**a**) LPAP proteoforms from resting cells. (**b**, **c**) Comparison of LPAP proteoforms derived from resting and PMA-activated cells. (**d**) Pro-Q Diamond Phosphoprotein Gel staining of LPAP phosphoforms after IP and 18% SDS–PAGE. (**e**, **f**) LPAP proteoforms resolved by Mn^2+^–Phos-tag SDS–PAGE. Specificity of LPAP bands was confirmed by WB (lower panels). (**g**) LPAP proteoforms resolved by Zn^2+^–Phos-tag SDS–PAGE. WB result is shown.

In 12% SDS–PAGE, LPAP migrated as a single band with the apparent MW of 32 kDa (Figure 2d). However, this band split into two bands of 32 and 29 kDa on high percentage low-porosity 18% SDS–PAGE (Figures 3a and b). The lower band of 29 kDa was clearly detected for CEM, HUT78, Jurkat and YT cells, but was relatively faint for Raji and KG1a cells, and LPAP ectopically expressed in HEK293 cells was associated with only the upper band. After the treatment of LPAP immunoprecipitates with phosphatase CIP, the lower band disappeared, and the intensity of the 32 kDa band became stronger.

The activation of cells with PMA led to a redistribution in the band intensities (Figure 3b). The lower band of LPAP from activated CEM and YT cells disappeared, whereas the intensity of the upper band increased. Upon the activation of Jurkat cells, both the upper and the lower bands of LPAP disappeared, and the middle band at 31 kDa appeared. Similar to LPAP from resting cells, only one band of 32 kDa was detected for the CIP-treated protein from activated cells (Figure 3c).

These experiments demonstrated that the major band of 32 kDa corresponded to the unphosphorylated LPAP, and the downward shifted band represented the phosphorylated form of LPAP. Nevertheless, both bands were detected using Pro-Q Diamond phosphoprotein gel stain (Figure 3d). This finding showed that the band of 32 kDa contained not only the unphosphorylated form but also a phosphoform, which did not have the mobility shift on SDS–PAGE.

SDS–PAGE mobility of a protein depends directly on its capacity to bind SDS. Phosphorylation can change this capacity, and as a result, phosphorylation-dependent mobility shifts of proteins can appear.^14^ However, only some phosphoproteins and phosphorylation sites are subjected to this phenomenon. In our case, one phosphoform of LPAP had a downward shifted band detectable only on a low-porosity gel. Based on the results of Pro-Q Diamond staining, we supposed that LPAP could have other phosphoforms that were not resolved by SDS–PAGE.

Phosphate-affinity SDS–PAGE with Phos-tag reagent is another method that enables the detection of phosphoforms of a protein.^15^ We examined whether this method could be applied to reveal additional phosphorylation states of LPAP. Using Mn^2+^-Phos-tag SDS–PAGE, we were able to resolve two bands of LPAP (Figure 3e). In contrast to the conventional SDS–PAGE, CIP treatment of LPAP caused the upper band to disappear, which proved that this band corresponded to phosphorylated LPAP. Furthermore, LPAP from activated cells contained only the lower band (Figure 3f). Thus, the upper band on Mn^2+^-Phos-tag SDS–PAGE behaved similarly to the lower band on the conventional SDS–PAGE. Although it could be inferred that they represented the same phosphoform of LPAP, experiments with alanine substitution mutants showed that they included different LPAP phosphoforms (unpublished observation).

The substitution of Mn^2+^ for Zn^2+^ and the application of the neutral-pH gel system in phosphate-affinity SDS–PAGE can improve the resolution of protein-phosphorylated forms.^16^ When we separated LPAP on Zn^2+^-Phos-tag SDS–PAGE, we detected three bands with large mobility differences, and two upper bands disappeared after the dephosphorylation of LPAP (Figure 3g). Thus, LPAP existed as at least three proteoforms, two of which were phosphorylated.

### LPAP proteoforms detected by 2D electrophoresis

To be separated on Phos-tag SDS–PAGE, phosphoforms of a protein should have phosphate groups accessible for the affinity interaction with Phos-tag.^17^ As a result, some phosphoforms with hidden phosphorylation sites do not show a delay in their migration. We hypothesized that LPAP could have such phosphoforms.

As phosphorylation changes the charge of a protein, the most direct method to determine protein phosphorylation states is 2D electrophoresis.^18^ In this method, proteins are separated in the first dimension by isoelectric focusing according to their isoelectric point (pI) and then in the second dimension by SDS–PAGE based on their molecular mass.

**Figure 4:**
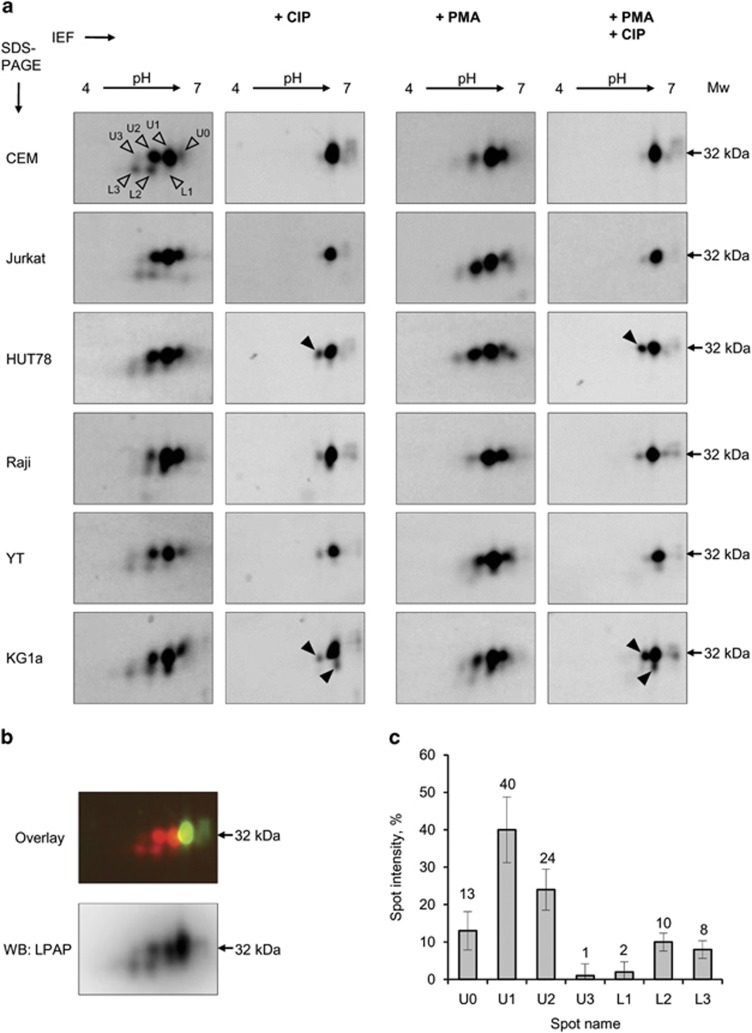
Comparison of LPAP proteoforms from various cell types detected by 2D-PAGE. (**a**) Resting (two left columns) and PMA-activated (two right columns) cells were labeled with Cy5 or Cy3 and lysed. Immunoprecipitated Cy3-labeled LPAP was treated with CIP (indicated as +CIP), whereas Cy5-labeled LPAP was left untreated. Samples of untreated (Cy5) and dephosphorylated (Cy3) LPAP were mixed, run on 2D-PAGE and visualized with a fluorescent gel scanner. A magnified view of the LPAP 2D region is presented. Open arrowheads indicate spots of LPAP detected in CIP-untreated resting CEM cells. Filled arrowheads show the protein spots that did not disappear after dephosphorylation with CIP. (**b**) Images of untreated (Cy5) and dephosphorylated (Cy3) samples from the resting CEM cells were overlaid (upper panel). Specificities of spots for LPAP protein were confirmed by western blotting (low panel). (**c**) Graph of spot intensities±s.d. (*n*=10) for resting CEM cells. Representative images of gels are demonstrated.

The analysis of LPAP proteoforms from CEM cells by 2D electrophoresis revealed the presence of at least seven spots in the pI range between 4.2 and 4.6 (Figure 4a, first column, upper panel). These spots lay in two trains shifted from each other on the molecular mass axis. The spots in both trains differed by ~0.07 pI units. We named the spots from the most alkaline to the most acidic as U0–U3 and L1–L3 for the upper and the lower trains, respectively (Figure 4a, spots indicated by open arrowheads). The spots U0–U2 and L2–L3 were detected in all experiments, whereas the spots U3 and L1 had a very low intensity and were visible only on some gels. Using western blotting, we confirmed that these spots belonged to LPAP, and no other spots on the gels contained our protein (Figure 4b, lower panel).

To determine which spots corresponded to phosphorylated LPAP, we compared the spot patterns of LPAP from CEM cells before and after the phosphatase treatment. Both CIP-treated and -untreated samples were mixed and subjected to 2D difference gel electrophoresis (2D-DIGE), which eliminated the problem of gel-to-gel variations.^19^ We found that the dephosphorylated LPAP sample contained only one high-intensity spot (Figure 4a, second column, upper panel). By overlaying the images of the untreated and the dephosphorylated samples, it was shown that the single spot from the dephosphorylated sample exactly matched the most alkaline spot U0 from the untreated sample (Figure 4b, upper panel). Thus, the spot U0 corresponded to the unphosphorylated proteoform of LPAP, whereas the spots U1, U2 and U3 represented mono-, di- and tri-phosphorylated forms. Apparently, the lower spot train of LPAP was connected with a phosphorylation site, which not only caused the change in pI but also led to an increased electrophoretic mobility.

Quantitative analysis of LPAP spots showed that the spots U1 and U2 accounted for 40% and 24% of the protein, respectively (Figure 4c). Thus, the largest proportion of LPAP from CEM cells existed, presumably, as mono- and di-phosphorylated forms.

Next, we compared the 2D-PAGE spot patterns of LPAP from several cell lines, including T- (CEM, Jurkat and HUT78), B- (Raji), NK (YT) and myeloid cells (KG1a). All cell lines showed relatively similar spot composition, but they differed in the distribution of the proteins between these spots. In particular, almost all of the LPAP from B cells—Raji (Figure 4a) and Ramos (data not shown)—lay in the spots U0 and U1, whereas the spots U1 and U2 were the most intense for LPAP from the lines of other cell types, including CEM and YT.

After the stimulation of cells with PMA, the number of spots decreased (Figure 4a, third column). In CEM and Raji cells, the lower train disappeared, and the upper train reduced to three spots: intensive U0 and U1 and faint U2. In KG1a and YT cells, the spot U2 was more pronounced, and the spot L1 was also detectable. Another spot pattern was observed for LPAP from Jurkat and HUT78 cells. Whereas the lower train spots were missing, the spots U1–U3 from the upper train shifted gradually to a lower MW in the second dimension. The spot U0 still corresponded to the dephosphorylated form of LPAP from all cell lines.

It should be noted that some signals of LPAP were retained in the spots U1 and L1 after the dephosphorylation with CIP. We observed this action most clearly in both the resting and PMA-activated HUT78 and KG1a cells (Figure 4a, spots indicated by filled arrowheads). These spots could not be removed by an increase in the CIP concentration by four times; therefore, we supposed that they contained not only phosphoforms but also some forms of LPAP with other post-translational modifications.

**Figure 5:**
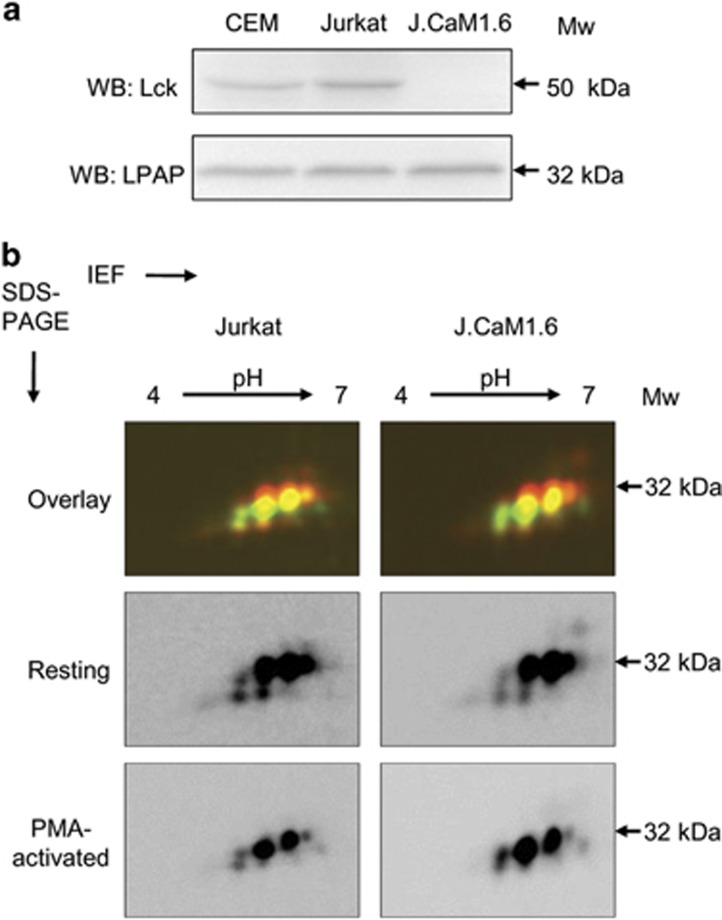
LPAP phosphorylation in Lck-deficient Jurkat cell line. (**a**) Straight WB demonstrating the expression of Lck (upper panel) and LPAP (lower panel) in cells. (**b**) 2D-DIGE comparison of LPAP proteoforms in wild-type Jurkat cells (left row) and Lck-deficient subline J.Cam1.6 (right row). Resting cells (middle panel) were labeled with Cy5, and PMA-stimulated cells (lower panel) were labeled with Cy3. Then, the cells were lysed, and LPAP was immunoprecipitated with anti-LPAP mAb. Samples from resting (Cy5) and PMA-stimulated (Cy3) cells were mixed and compared by 2D-DIGE. Upper panel represents the overlay of resting and PMA-stimulated samples. Gels and blots are representative of three independent experiments.

Among the various cellular kinases, Lck kinase was the first that could potentially phosphorylate LPAP because both molecules were detected as members of a supramolecular complex containing CD45, CD4, Lck and LPAP.^9, 20^ To test this possibility, we used a derivative of the Jurkat cell line deficient for Lck, J.CaM1.6 (Figure 5a). As both Jurkat and J.CaM1.6 cells demonstrated identical spot patterns in the resting and activated states (Figure 5b), we concluded that Lck was not essential for LPAP phosphorylation.

## DISCUSSION

LPAP is a transmembrane protein that specifically marks the population of lymphocytes, and therefore, it can be formally included in the cluster of differentiation (CD) nomenclature.^21^ However, this nomenclature requires that a protein must be detected on the surface of the primary cells with the mAbs that are characterized through the workshop process. Nevertheless, there are some exceptions to this rule. Thus, toll-like receptor TLR7/CD287 is localized within endosomal compartments, and CD3 zeta chain/CD247 has a very short extracellular domain. To detect these antigens with antibodies, plasma membrane permeabilization is needed. Likewise, staining for LPAP requires cell permeabilization. In previous studies, the expression of LPAP was estimated at the level of mRNA^10^ and with polyclonal antibodies.^1, 11^ In the current study, we characterized new anti-LPAP mAbs generated in our laboratory. These mAbs effectively detected LPAP via widely used techniques, including immunofluorescence, immunoblotting, IP and immunohistochemical staining of formalin-fixed, paraffin-embedded tissues.

Both our results and reported data demonstrate that LPAP expression is detected in various populations of lymphocytes (that is, T, B and NK cells), as well as in some myeloid cells.^1, 10, 11^ The level of LPAP expression generally correlates with the expression level of CD45. Nevertheless, CD45 has a more broad distribution and can be expressed without LPAP in some myeloid cells. However, we did not find any CD45-negative cell line that would constitutively express LPAP. This result is probably because LPAP is quickly degraded in cells if CD45 is absent.^1^

However, the presence of CD45 is not an absolute requirement for LPAP expression. Thus, human embryonic kidney cells HEK293 transfected with the expression plasmid that encodes LPAP expressed high levels of LPAP in the absence of CD45. Interestingly, murine LPAP was also detected in the CD45-negative T-cell line SAKRTLS 12.1.^10^

LPAP likely exists in cells in various proteoforms that can represent phosphorylated variants of the protein. As mentioned earlier,^1^ the resting Jurkat cells express two forms of LPAP that have slightly distinct electrophoretic mobilities, LPAP32 and LPAP29. After the activation with PMA, these forms are converted to two other forms, LPAP30 and LPAP31. In our report, we demonstrated that LPAP has more phosphoforms than previously described; in addition to the unphosphorylated form, there are at least six phosphoforms of LPAP that we detected on 2D-PAGE.

Our experimental results have shown that LPAP proteoforms from the upper train detected on 2D-PAGE have pI values of 4.54, 4.47 and 4.40. Using ProMoST software,^22^ we calculated that unphosphorylated, mono-, di- and tri-phosphorylated forms of LPAP should have pI values of ~4.40, 4.34, 4.28 and 4.22, respectively. These estimates were slightly different from our experimental data. However, the pI shift between protein spots determined experimentally (0.07) was very close to the pI shift predicted by the databases (0.06). Thus, the calculation confirms that the upper spot train corresponds to the mono-, di- and tri-phosphorylated forms of LPAP. The lower spot train is likely representative of LPAP with another phosphorylation site, in which modification alters not only the molecular charge but also the conformation of the protein and its ability to bind SDS. In other words, to interpret the 2D pattern of LPAP proteoform distribution, we have to assume that LPAP has at least four sites of phosphorylation. The high number of phosphorylation sites in such a small molecule suggests that LPAP has a high functional potential in the regulation of cell activation status. It is believed that multisite phosphorylation can create a high threshold level and switch-like response processes in the cell activation that it regulates.^23, 24^

LPAP is abundantly expressed in lymphoid cells,^25^ but its function remains elusive. As the major molecular partner of LPAP is CD45 phosphatase, many researchers consider that the function of LPAP is only the regulation of phosphatase activity in lymphocytes. The contradictory data on the involvement of LPAP in CD45-regulated receptor signaling have been reported using CD45-AP-deficient mice. Some groups denied the role of LPAP in receptor signaling,^5, 6^ whereas others have claimed that LPAP enhanced signal transduction from T-cell and B-cell receptors.^7^ The most likely relevant assumption is that CD45-AP influences T-cell activation status only when receptor signaling is triggered by low-potency antigens.^26^ Under these conditions, the phosphatase activity of CD45 in CD45-AP-deficient mice was reduced compared with their wt counterparts.^27^ The other potential function of LPAP as an adapter molecule is to bridge CD45 to other molecules involved in signal transduction.^8^ For example, LPAP can interact with the kinase Lck and recruit it to the CD45 molecular complex.^9^ After the antigenic exposure of T or B cells, LPAP binds to another protein tyrosine kinase ZAP-70.^28^ The formation of the LPAP-CD45 molecular complex can also control the subcellular distribution of CD45 and consequently the activity of the phosphatase.^10^ The conceptually different view regarding LPAP function lies in the involvement of LPAP in B-cell development and homeostasis. This idea is supported by the findings of increased cellularity^6, 25^ and altered numbers and percentage of transitional and mature B-cell subsets^25^ in lymph nodes obtained from LPAP-null mice. In summary, although scientists have made efforts to elucidate the biological role of LPAP, the functionality of this small adapter molecule remains mostly unknown.

We believe that the identification of kinases and phosphatases, which are involved in the phosphorylation/dephosphorylation of LPAP, will reveal the function of LPAP. It is tempting to speculate that Lck kinase, which has been reported to associate with LPAP,^9, 20^ might be involved in LPAP phosphorylation. However, our data do not support this hypothesis because J.CaM1.6, a Lck-deficient Jurkat cell line, expressed identical phosphoforms of LPAP that we registered on 2D-PAGE compared with wt Jurkat cells.

In conclusion, using mAbs and the combination of protein analysis tools, we determined that LPAP is a multiple phosphorylated protein. LPAP is present in cells in at least six phosphoforms. Currently, we are in the process of identifying the amino acids in LPAP that are modified by phosphorylation. Accordingly, we generated CEM T-cell lines with the stable expression of LPAP bearing a mutation at one of the potential phosphorylation sites. We suggest that identifying all phosphorylation sites of LPAP and understanding the role of transitions between various protein phosphoforms will increase our knowledge of the function of LPAP.

## METHODS

### Cells and mAbs

Human cell lines (Jurkat, JCam1.6, CEM, HUT78, MOLT-4, HPB-ALL, Raji, DAUDI, IM-9, Ramos, NALM-6, CCRF-SB, U266, RPMI 1788, RPMI 8226, YT, U-937, HL-60, THP-1, KG1a and K-562) were maintained in RPMI 1640 medium or Dulbecco's Modified Eagle's Medium, supplemented with 10% fetal bovine serum and 24 μg ml^−1^ of Gentamicin (all Paneko, Moscow, Russia). PMA (Sigma, St. Louis, MO, USA) activation was performed for 4 h at 37 °C in complete RPMI 1640 medium with 8 nm PMA.

Peripheral blood mononuclear cells from healthy donors were isolated by centrifugation on the Ficoll/Paque (GE Healthcare, Pittsburgh, PA, USA) density gradient. To generate monocyte-derived dendritic cells,^29^ peripheral blood mononuclear cell were adhered to the plastic surface and cultivated in complete RPMI 1640 medium supplemented with 100 ng ml^−1^ granulocyte-macrophage colony-stimulating factor and 20 ng ml^−1^ interleukin-4 (BD Biosciences, San Diego, CA, USA). Immature dendritic cells were stimulated on day 5 with 100 ng ml^−1^ lipopolysaccharide (Sigma) and 2 days after immature and stimulated dendritic cells were harvested.

Anti-LPAP mAbs CL3 (immunoglobulin G1; IgG1), CL4 (IgG1), CL7 (IgG2a) and anti-CD45 mAb LT45 (IgG2a) were produced in our laboratory.^12, 30^ The mAb LCK-01 (IgG1, anti-Lck) was purchased from Exbio (Praha, Czech Republic).

### Plasmids, transfection and infection

The cloning of human LPAP into pCMVpA expression plasmids and lentiviral vector pUCHR IRES GFP have been described previously.^12, 31^ To knockdown CD45 expression, we used pGIPD mir30-based lentiviral vector from Open Biosystems (Huntsville, AL, USA). Two pGIPD plasmids with target sequences for PTPRC (CD45) open reading frame starting at the positions 3136 and for the phosphoprotein of human respiratory syncytial virus (as a control) were constructed as described.^32^ To generate cell lines with the stable expression of LPAP or short hairpin RNA, HEK293 cells in 6-cm dishes were transiently cotransfected with three plasmids: transfer vector pUCHR (or pGIPD), HIV-1 packaging plasmid pCMV-8.2R and plasmid pCMV-VSVG, expressing Env G from vesicular stomatitis virus, in amounts of 3, 2 and 0.5 μg, respectively. Cells were transfected with Lipofectamine 2000 reagent (Invitrogen, Eugene, OR, USA). Two days post transfection, HEK293 cell-derived virus like particles were collected, clarified through 0.45 μm pore size filter and used to infect target cells. Three days after infection, a pool of cell that stably express short hairpin RNA or LPAP were selected by growing cells for 2 weeks in the presence of 1 μg ml^−1^ Puromycin (Invitrogen) or 800 μg ml^−1^ Geneticin (Gibco, Grand Island, NY, USA), respectively.

### Immunofluorescence and immunohistochemical analysis

For intracellular staining, cells were washed with phosphate-buffered saline (PBS) and fixed with 1% paraformaldehyde (Sigma) in PBS for 5 min. Then, cells were permeabilized in PBS containing 0.1% saponin (Sigma) and 5% (w/v) nonfat milk for 30 min. Cells were stained using mAbs, followed by secondary Alexa 488 conjugated antibodies. The stained cells were analyzed using a FACScan flow cytometer (BD Biosciences). The levels of fluorescence were measured and expressed as a mean fluorescence intensity. The cells treated only with the secondary Abs were used as a negative control.

For confocal microscopy, cells were washed with PBS and added to coverslips precoated with 0.1% poly-l-lysine, fixed in 4% paraformaldehyde, and stained with antibodies as described above for flow cytometry analysis. The endoplasmic reticulum was stained with tetramethylrhodamine-labeled concanavalin A (Invitrogen) in permeabilization solution without serum.^33^ After washing, coverslips were transferred to the slides and maintained in fluorescent mounting medium (DakoCytomation, Glostrup, Denmark). Slides were analyzed using a Nikon Eclipse Ti confocal microscope (Nikon Corp., Tokyo, Japan). Green and red fluorescence were recorded using a × 100 objective lens.

Formalin-fixed paraffin-embedded tissue specimens of the thymus and spleen were cut into 5 μm sections. Sections were deparaffinized and processed for immunohistochemistry using an Elite ABC Peroxidase Kit (Vector Labs, Burlingame, CA, USA) following the manufacturer's instructions. Sections were stained with diaminobenzidine substrate.

### Fluorescent immunoprecipitation analysis

The labeling of cells with amino reactive fluorescent dyes Cy3 or Cy5 (BioDye, Moscow, Russia) was performed as described previously.^30^ Briefly, 2 × 10^7^ cells were washed with PBS (pH 7.4) two times and resuspended in 1 ml of PBS. Then, 30 μl of cyanine succinimidyl ester stock solution in dimethyl sulfoxide (10 mg ml^−1^) was added to the cells and incubated for 20 min. Unreacted dye was removed by washing cells with PBS two times. Cells were lysed in 1 ml of buffer containing 20 mm Tris-HCl (pH 8.0), 1% Triton X-100, 150 mm NaCl, 5 mm EDTA and 1 mm phenylmethylsulfonyl fluoride, 10 mm NaF and 1 mm Na_3_VO_4_ for 30 min at 4 °C (all reagents were purchased from Sigma). In co-IP experiments for cell lysis instead of Triton X-100, we used mild detergent Brij97. Cell debris was pelleted at 20 000 × * g* for 15 min at 4 °C. Cell lysates were cleared overnight at 4 °C by rotation with normal mouse IgG covalently linked to CNBr-Sepharose. Precipitations of precleared lysates with specific Abs were carried out by using 30 μl 25% AffiGel Hz hydrazide agarose beads (Bio-Rad, Hercules, CA, USA) coupled with anti-LPAP mAb CL7 or anti-CD45 mAb LT45. Samples were precipitated under rotation at 4 °C for 2 h. Then, the beads were washed four times in the lysis buffer. The proteins were eluted by heating the beads in the SDS sample buffer at 80 °C for 5 min or in isoelectric focusing sample buffer containing 7 m urea, 2 m thiourea, 2% Triton X-100, 2% ampholytes, pH 3–10, and 100 mm dithiothreitol at 28 °C for 2 h.

### Dephosphorylation

LPAP obtained by IP with AffiGel-CL7 beads was transferred into the buffer containing 50 mm Tris-HCl (pH 7.6); 10 mm MgCl_2_; 100 mm NaCl; 1 mm dithiothreitol. Ten units of CIP (SibEnzyme, Novosibirsk, Russia) were added to 50 μl of this solution and incubated 1 h at 37 °C. Samples were resuspended in electrophoresis sample buffer and analyzed by gel electrophoresis.

### Electrophoresis

Eluted samples were subjected to Laemmli SDS–PAGE, Phos-tag SDS–PAGE, or 2D electrophoresis. Laemmli SDS–PAGE was performed with 12% or 18% polyacrylamide gels under reducing conditions. Phos-tag SDS–PAGE^15, 16, 17^ was performed with 10% polyacrylamide gels containing 50 μm Phos-tag acrylamide (Wako, Osaka, Japan) and 100 μm MnCl_2_ or ZnCl_2_. For 2D-DIGE samples labeled with Cy3 and Cy5 were mixed and loaded on 7 cm Immobiline DryStrip pH 4–7 (GE Healthcare) and separated in the first dimension using an isoelectric focusing system Ettan IPGphor 3 (GE Healthcare) as follows: 300 V for 30 min, 1000 V for 30 min (gradient), and 5000 V for 1.5 h (gradient), 5000 V for 30 min. After isoelectric focusing, strips were equilibrated in reducing solution (50 mm Tris-HCl pH 6.8, 6 m urea, 30% glycerol, 2% SDS) containing 1% dithiothreitol for 15 min and then in the same buffer containing 5% iodoacetamide instead of dithiothreitol for 15 min. 2D-PAGE was performed using 18% resolving gels at 180 V for 3 h. Gels after one-dimensional or 2D electrophoresis were visualized using an Amersham Imager 600RGB (GE Healthcare).

### Pro-Q diamond staining

Gels after SDS–PAGE were fixed in solution containing 50% (v/v) ethanol and 10% (v/v) acetic acid. On the following day, the gels were washed three times with ddH_2_O for 10 min and stained with Pro-Q Diamond phosphoprotein stain (Invitrogen) for 90 min. The gels were washed three times with ddH_2_O for 10 min, destained twice for 30 min in 50 mm sodium acetate, pH 4.0, containing 20% (v/v) acetonitrile and finally washed with ddH_2_O. Fluorescent bands on the gel were visualized with an Amersham Imager 600RGB (GE Healthcare).

### Immunoblotting

Proteins from the gel were transferred by blotting onto PVDF membrane (Millipore, Billerica, MA, USA) by semi-dry method for 25 min using Mini Trans-Blot apparatus (Bio-Rad). Membranes were blocked overnight at 4 °C with 5% (w/v) nonfat milk in PBS containing 0.02% Tween 20, probed with primary antibodies, washed with PBS-Tween and developed with horseradish peroxidase-conjugated secondary antibodies (Cell Signaling, Danvers, MA, USA). Blots were washed again, and immunoreactive bands were detected with Immobilon Western reagent (Millipore) on a Molecular Imager ChemiDoc XRS (Bio-Rad).

### Statistical analysis

*P*-values were calculated by paired Student's *t*-test, and significance was defined as *P*<0.05. Data are reported as a mean±s.d. as indicated. The correlation between LPAP and CD45 expression was calculated using Pearson's correlation coefficient (*r*).

## Figures and Tables

**Table 1:** Reactivity of anti-LPAP and anti-CD45 mAb with human primary and transformed cells

	*LPAP*	*CD45*
*Transfectants*		
HEK293-mock	−	−
HEK293-LPAP	+++	−
		
*Primary cells*		
Peripheral blood lymphocytes	+++	+++
Peripheral blood monocytes	−	+++
Peripheral blood neutrophils	−	++
Thymocytes	+++	++
Monocyte-derived immature dendritic cells	−	
Monocyte-derived stimulated dendritic cells	−	
Erythrocytes	−	−
		
*Cell lines*		
Jurkat (T-cell leukemia)	+++	+++
CEM (acute T lymphoblastic leukemia)	+++	+++
HUT78 (T cell lymphoma)	+++	+++
MOLT-4 (acute lymphoblastic leukemia)	++	+
HPB-ALL (T cell lymphoma)	−	−
DAUDI (Burkitt's lymphoma)	++	+++
Raji (Burkitt's lymphoma)	++	+++
Ramos (Burkitt's lymphoma)	++	+++
CCRF-SB (acute lymphoblastic leukemia)	+	++
IM-9 (multiple myeloma)	++	+++
RPMI 1788 (B lymphocytes)	−	+
Reh (pro-B leukemia)	++	+++
NALM-6 (pro-B leukemia)	−	−
RPMI 8266 (myeloma)	−	−
U266 (myeloma)	−	++
YT (NK leukemia)	+++	+++
HL-60 (AML-M2)	+	++
THP-1 (AML-M5)	−	++
MONO-MAC-6 (AML-M5)	−	+++
U-937 (histiocytic lymphoma)	−	++
K-562 (CML-erythroblast)	−	+++
		
*Cell lines (solid tumors)*		
Hep G2 (hepatocellular carcinoma)	−	−
MCF7 (adenocarcinoma)	−	−

Abbreviation: MFI, mean fluorescence intensity.

Scores: (−) MFI<50; (+) 50<MFI<100; (++) 100<MFI<200; (+++) 200<MFI.
